# Real-time PCR assay for discrimination of *Plasmodium ovale curtisi* and *Plasmodium ovale wallikeri* in the Ivory Coast and in the Comoros Islands

**DOI:** 10.1186/1475-2875-11-307

**Published:** 2012-09-04

**Authors:** Frédérique Bauffe, Jérôme Desplans, Christophe Fraisier, Daniel Parzy

**Affiliations:** 1 UMR-MD3, Aix-Marseille Université, Institut de recherche Biomédicale des Armées, Marseille, France; 2 WHIDIAG, 100 route des houillères, Meyreuil, 13590, France

**Keywords:** *Plasmodium ovale curtisi*, *Plasmodium ovale wallikeri*, Real-time TaqMan® PCR, Diagnostic, Sympatry

## Abstract

**Background:**

*Plasmodium ovale* is one of the five malaria species infecting humans. Recent data have shown that the name of this neglected species masks two distinct genotypes also called *curtisi* and *wallikeri*. Some authors show that these species could be sympatric. These two subspecies are not differentiated by microscopy techniques and malaria rapid diagnostic tests. This diagnostic defect is the result of low parasitaemia, antigenic polymorphism and absence of antibodies performance and requires the use of sequencing techniques. An accurate and easy discrimination detection method is necessary.

**Methods:**

A new molecular assay was developed to easily identify the two genotypes of *P. ovale*. This tool allowed the study of 90 blood samples containing *P. ovale*, confirmed by molecular biology techniques, which were obtained from patients with imported malaria.

**Results:**

The new marker was validated on well genotyped samples. The genotype of 90 *P. ovale* samples mainly imported from the Ivory Coast and the Comoros Islands was easily and quickly realized. The distribution of the two subspecies was described with a significant number of samples and showed that the two genotypes were present in the studied countries.

**Conclusion:**

This work confirms the presence of the two species in the same country for the first time, in the Ivory Coast and the Comoros Islands. A better genotyping of *P. ovale* types may improve a better characterization of the clinical pathophysiology for each*.*

## Background

*Plasmodium ovale* is the third species, in case number, of agents causing human malaria. It was first described in 1922 [1]. It has been reported all over the subtropical continents [2], but is commonly found in tropical Africa, New Guinea, Indonesia and Philippines. Its prevalence rate, probably underestimated, ranged from 0.5 to 10.5% of all malaria cases in 2005 [3]. It is estimated that about 15 million people are infected by this parasite [4]. Its importance is growing, due to the decrease in the incidence of *Plasmodium falciparum* and the potential interactions with the other malaria infections.

*Plasmodium ovale* is still sensitive to all anti-malarial drugs, and it shares the particularity with *Plasmodium vivax* to form latent stage in the liver, referred to as hypnozoite. This original behaviour is responsible for the late relapse of the parasite, with new febrile access without recent events of exposure. These febrile episodes may occur in intervals of time that can reach several months or years [3,5] and which require a specific treatment with primaquine for complete clearance, obtained after several cures. Severe cases have rarely been described, but could occur nonetheless [6,7].

*Plasmodium ovale* diagnosis is difficult for several reasons, particularly due to a frequent low level of parasitaemia [8], and the errors of microscopic diagnosis with other malarial species*.* The low level of parasitaemia is a disadvantage for the RDT detection. The lack of sensitivity and specificity of rapid diagnostic tests (RDT), as no specific monoclonal antibody yet available leads to poor *P. ovale* identification. Furthermore, the Pan signal is often ineffective for its detection. The failures of *P. ovale* detection by RDT are well documented [9,10]. Genetic variations based on *P. ovale* LDH polymorphism could be involved in RDTs failure [11]. To overcome this difficulty, diagnostic methods using molecular biology techniques have been developed. However, few target genes are known because the total sequence of the *P. ovale* genome is not yet available.

Species-specific sequences within genes encoding small subunit ribosomal RNA (*SSU rRNA*) have been used as primers and have allowed discrimination between different malaria parasites through sequencing [12]. Different sets of primers were successively designed to improve the diagnosis, which illustrated the problem caused by the two genotypes for the diagnostic of the *P. ovale* subspecies [13]. Rougemont *et al.* developed a real-time PCR (RT-PCR) assay, which can separately distinguish the four major *Plasmodium* species [14]. Recently, another RT-PCR method was developed to identify the rare species from a mixed infection [15,16].

Complementary molecular investigations allowed the identification of a polymorphism within the *P. ovale* sequences. Species identification by 18S RNA sequencing split the samples into two groups, classic/variant type respectively named *Plasmodium ovale curtisi* and *Plasmodium ovale wallikeri* by Sutherland *et al.*[4,17,18]. Other investigations have confirmed that by the sequencing of nine other target genes [4,11,18-22] whose genes encoding the lactate dehydrogenase (LDH) and the ookinete surface protein. Further studies with a conventional PCR or SYBR-Green real-time PCR method confirm the existence of the simultaneous presence of two subspecies in Africa [21].

A molecular marker based on hydrolysis probe technology was developed to quickly distinguish the two genotypes. This technique was used to study samples from countries for which less information were available, the Ivory Coast and the Comoros Islands, and the link between RDTs failure and genotyping group was studied.

## Methods

### Samples

*Plasmodium ovale* samples were selected among previously characterized blood samples collected from 2005 to 2010 from patients hospitalized in France and infected with imported malaria. The samples, collected in EDTA or ACD tubes, were stored at 4°C and were received within 24 to 48 hours. Thin blood films and RDTs were immediately performed. After centrifugation, sera and the red blood cells pellet were separated and stored at −20°C until analysis. For each sample, identification of the parasite species was performed by microscopy, immunochromatography and molecular biology. A total of 90 samples containing *P. ovale* parasites were retained for further study.

### Species detection

Thin blood films were immediately stained by Giemsa to identify parasite species. They were read at least 20 min before to consider the smears as negative. RDT was performed on all the symptomatic samples received. These tests allowed *Plasmodium* species to be quickly identified. Normally all tests used should detect *P. ovale* species in a pan-species result, according to the manufacturer‘s instruction for use. From 2005 to 2008, Now malaria® (Binax, UK) was used, replaced by Core Malaria® (Core Diagnostics Ltd, UK) or Palutop® (Alldiag, Strasbourg, France) since 2009. These RDTs detect ALDOLASE and LDH antigen respectively in the *P. ovale* samples. All these tests were performed according to the manufacturers’ instructions except for blood pipetting with a micropipette (10 μl) for more reproducible results.

### Molecular *Plasmodium* species detection

Parasite genomic DNA templates were isolated from 200 μl of frozen red blood cells pellet using the DNA isolation KIT (QIA amp DNA mini kit, QIAgen, Hilden), according to the manufacturer's instructions. The *Plasmodium* species detection of *P. ovale* was performed with primers Pos25S and Pos25R [GenBank:AB074973] (Table 1) using SYBR green qPCR as previously described [15]. Since 2010, malaria species samples were determined by real-time PCR using TaqMan probes and a set of primers specific to the same target genes (Table 1). 

**Table 1:** Primers and probes used for sequencing and TqPCR quantification

**Type**	**Primer/Probe**	**5’ Fluorophore**	**Sequence**	**3’ Quencher**
sp	Pf B		GGCAAATAACTTTATCATAGAATTGAC	
sp	Pf F		TTTATGTATTGGTATAACATTCGG	
sp	Pv1087f		GTGGCCGCCTTTTTGCT	
sp	Pv1196r		CCTCCCTGAAACAAGTCATCG	
sp	PovS		CCAAGCCCAGATAATAAGGAAGGT	
sp	PovR		TTCGTGCACTTCAACTTACATTCAGT	
sp	PmalS		GGAGGAATGGTCACCATGTAGTGT	
sp	PmalA		CAAATTTCAGTTTCAAGGTCACTTAA	
sp	pPf	FAM	TACACTACCAACACATGGGGCTACAAGAGGT	BHQ1
sp	pPv	HEX	CATCTACGTGGACAACGGGCTCAACA	BHQ1
sp	pPo	FAM	TTATTGTCCTCTGGGTTTGGAACTTTGCC	BHQ1
sp	pPmal	HEX	ATTTTTTGCATCAACCTTTCTTCTAGCCC	BHQ1
C/W	POF		ATAAACTATGCCGACTAGGTT	
C/W	POR		ACTTTGATTTCTCATAAGGTACT	
C/W	pPOW	HEX	AATTCCTTTTGGAAATTTCTTAGATTG	BHQ1
C/W	pPOC	FAM	TTCCTTTCGGGGAAATTTCTTAGA	BHQ1
seq	P1F-Up		TCCATTAATCAAGAACGAAAGTTAAG	
seq	18S R		TAATGATCCTTCCGCAGGTTCACC	
seq	LDH ov D21		GTTCTCGTTGGTCAGGAATGATA	
seq	LDH ov C915		GGCATCATCAAACATCTTCTTTTCT	

For species detection (sp), sequencing (seq) or *P .ovale* genotyping (C for *P. o. curtisi* /W for *P. o. wallikeri*).

### *Plasmodium ovale* subspecies detection

The detection was carried out by sequencing the *SSU rRNA* and/or *Po-ldh*. The primers P1F-Up and 18S R (Table 1) from Win [19] bound the *SSU rRNA* sequence. *Po-ldh* gene polymorphism was established by sequencing the central fragment of the gene. Amplification was carried out with primers LDHovD21 and LDHovC915 designed at the extremities of the *Po-ldh* sequence [GenBank AY486058]. The number in the primer indicates the position of the 5' base from the coding start position of the *Pf-ldh* sequence [PlasmoDB: PF3D7_134900] (Table 1) [23].

The real-time TaqMan PCR (TqPCR) assays were performed in a final volume of 20 μl containing 5 μl of DNA, 1X of master mix (Light Cycler TaqMan master, Roche, Germany), 0.1 μM of probe and 0.8 μM of each primer (Eurogentec, Belgium). The cycle conditions used were as follows: one step at 95°C for 10 min, 45 cycles at 95°C for 10 sec and 60°C for 30 sec, and a final step at 40°C for 30 sec, with a constant ramp rate at 20°C/sec. Fluorescence was read at the end of each cycle on LightCycler® 2.0 device (Roche Applied Science, Mannheim, Germany).

A sequence of the *SSU rRNA* gene was also used to design a pair of primers named PoF and PoR and two probes (pPOC and pPOW) (Table 1) for the TqPCR analysis (Table 2). A Clustal [24] alignment was made on sequences from “malmai” [seq X99790] and “poc1” [AB182489] for *P. o. curtisi* and *P. o. wallikeri* forms, respectively [19]. Each probe is specific to one of the two *P. ovale* types and displays a distinct HEX or FAM fluorochromes, with readings at 530 and 560 nm for pPOW and pPOC, respectively. The primers, with a Tm of 60°C, were designed by Primer Express®software v2.0, and the probes, with a Tm of 10 degrees higher according to the TaqMan technology specification, were manually designed. The probes were designed to target a deletion at position 1158 in variant *SSU rRNA* sequence [19]. The TqPCR amplified fragment is 118 bases. The pPOW probe contains the deletion, present in all sequences of this type and the pPOC probe was designed in the same place in the classic sequence. 

**Table 2:** Genotype determination by sequencing and POCPOW assay

			**TqPCR**		
**Gene**	**Type**	**Sample (n = 70)**	**POC (n = 44)**	**POW (n = 26)**	**% similarity**
*SSU rRNA*	curtisi	14	14	0	100
*SSU rRNA*	wallikeri	9	0	9	100
LDH	curtisi	30	30	0	100
LDH	wallikeri	17	0	17	100

Samples were first identified by amplification of the *SSU rRNA* and *ldh* sequences, thanks to the primers displayed below. The results were compared with the TqPCR results and the similarity was calculated. Four *P. o. curtisi* samples and four *P. o. wallikeri* samples were sequenced for both *SSU rRNA* and *ldh* genes.

As positive control, DNA from well characterized samples of each *P. ovale* type was used. The efficiency of each reaction was assessed using 10-fold dilutions of the positive control (from 1/10 to 1/1000) in triplicate and the slope of standard curves generated by plotting graphs of genomic DNA concentrations *vs* Ct values was determined. The specificity was verified by using the four other human malaria species DNA (*P. falciparum, P. vivax, Plasmodium malariae* and *Plasmodium knowlesi*) and human DNA as target template.

## Results

### Samples

The selection of blood samples from imported cases of malaria patients mainly from the Ivory Coast and the Comoros Islands, has allowed the study of 90 *P. ovale* DNA samples. Species identification was previously performed by microscopy observations and RDTs. The molecular method was used to confirm the species diagnostic. However, these methods were not able to distinguish the *P. ovale* subtypes. This identification is possible by sequencing the *18S RNA* gene or several coding sequences previously published, but the delay to obtain the result is long [19].

### Marker set-up

This RT-PCR was set up on two reference samples, defined as “classic” (Poc) and “variant” (Pov), according to both LDH and *SSU rRNA* gene sequencing. The *SSU rRNA* sequence polymorphisms are broadly used references [12]. The *P. ovale* LDH (*PoLDH*) gene was selected since the corresponding protein is detected in RDT diagnosis and genetic variation in this protein was suggested as a possible cause for RDT failure.

The efficiency of primers (named PoF and PoR) and probes set (named pPOC for *P. o. curtisi* and pPOW for *P. o. wallikeri*) has been determined in a specific assay. The mean curve slope and coefficient correlation were respectively −3.18 and 0.997 for the *P. o. wallikeri* type, with corresponding mean reaction efficiency of 99.71% and for the *P. o. curtisi* form, the mean curve slope and coefficient of correlation were −3.28 and 0.997, respectively, with a corresponding mean reaction efficiency of 99.77%. The sensitivity was tested with parasitaemia dilutions of 0.0065% to 0.000065% (Ct of 27.16 and 33.35 respectively) for *P. o. wallikeri*, and 0, 01% to 0, 0001% (Ct of 27, 50 and 34, 00 respectively) for *P. o. curtisi*.

These new sets of primers and probes are very specific, and they did not cross with other malaria species or human DNA.

### Samples genotyping

The validation of these markers was made on 62 *P. ovale* samples, previously characterized either their *SSU rRNA* or *ldh* gene sequence (Table 2). The sequencing of the central part of the *ldh* gene distinguished the two subspecies thanks to the substitutions on the codon positions S143P, 1N68K, previously describes, and I204V for *P. o. curtisi* and *P. o. wallikeri*: respectively Additional file (1) [11,20].

**Figure 1:**
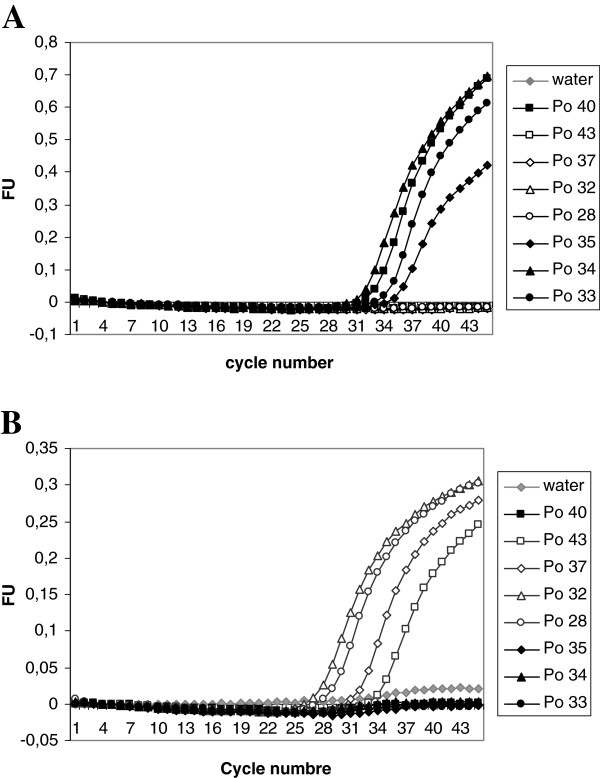
** Real-time amplification plot with POC marker (A) and POW marker (B) of three samples of each, *P. o. curtisi *(black) and *P. o. wallikeri *(white).** Water line is negative control (grey rhombus). Positive control (square). The X-axis: the cycle number and the Y-axis: the fluorescence arbitrary unit (FU). Detection was carried at 530 nm and at 560 nm for (**A**) and (**B**) respectively.

Among the 62 samples, the sequencing of the *ldh* and *SSU rRNA* genes has been done both for eight samples and the new marker validate these results (Figure 1A and 1B). The “POCPOW” markers were used to identify the subspecies of remaining *P. ovale* samples. For all the samples studied, 31 were determined as *P. o. wallikeri* types and 59 as *P. o. curtisi* types Additional file (2).

### Co-existence of *Plasmodium ovale* populations

The determination of the genotype gave the opportunity to study the country repartition of the two *P. ovale* subspecies. The used samples came from different countries, especially from Africa, with a predominant recruitment from Ivory Coast and the Comoros Islands. In general terms, the *P. o. curtisi* form is more widespread than the *P. o. wallikeri* in studied sample. The *P. o. curtisi* form represented 62.8% of samples and 37.1% belonging to the *P. o. wallikeri* form (Table 2). In the Ivory Coast, most of the samples were *P. o. curtisi* form (25/31 samples) whereas the majority of the samples from the Comoros Islands (11/18 samples) were of *P. o. wallikeri* form. This distribution is statistically different (Chi2 test; p = 0,031).

### Parasitaemia level

Easy identification of the two subtypes allowed investigation of the behaviour of the two genotypes. The parasitaemia was established and validated in 63 samples by an experienced microscopist. It was shown to be low ranging from to 0 to 0.5% (Additional file 2).

The two population statistical analysis with Wilcoxon test showed no parasitaemia level difference (Chi2 test p = 0.3353).

### RDT failure

Laboratory practice and literature reported that RDT failure is often observed with *P. ovale* samples. This RDT failure is currently hypothesized by the role of natural variability present in tested species, as well as by the low parasitaemia often encountered. It was observed a higher false negative rate for the *P. o. curtisi* than for the *P. o. wallikeri* (about 60% *vs* 43% respectively). However, due to the relatively small number of samples available for each RDT and lot used, the difference was not statistically significant. From these results, the three tests used have the same efficiency with ALDOLASE or LDH antigens detection. The false negative results rates for the detection of *P. o. curtisi* with RDTs using ALDOLASE or LDH antigens were 60% and 63% respectively. For the detection of *P. o. wallikeri*, the rate of false negative results was 43% no matter the antigen used by the tests. The positive results rates for the detection of *P. o. curtisi* with RDTs using ALDOLASE or LDH antigens were 40% and 37% respectively and for *P. o. wallikeri* detection, the positive results rates was 57% for the 2 antigens used.

The other factor commonly associated with RDT failure is low parasitaemia level. In this study, there is no influence of parasitaemia on RDT failure (Chi2 test p = 0.4108). The result of the RDT based on LDH antigen detection seems to be less affected by the low parasitaemia level than those detecting the aldolase antigen (Chi2 test p = 0.9843 and Chi2 test p = 0.0314 without the outlayer data of 0.5% respectively).

## Discussion

The single microscopic diagnostic had for a long time limited the description of human malaria to four species. Microscopic diagnostic is an insufficient method to accurately discriminate between the species [7,25], because of its limited resolution, the morphological similarity of certain *Plasmodium* species and, especially, the expertise of the microscopist. In the same way, *P. o. curtisi* and *P. o. wallikeri* were confused due to their morphology. Until recently, the molecular methods detected *P. ovale* without distinguishing the subspecies. The consequence is doubt in the epidemiological study due to a lack of tools in the rare species diagnostic, such as *P. ovale* and *P. malariae*, which are less known and in fact more difficult to be characterized [7]. A better understanding of the circulating malaria species in the environment could increase the success in the identification of the human infectious species. For that, molecular methods have been developed and now bring corresponding tools to specifically identify each species or subspecies.

In this study, a rapid method has been developed to distinguish the two *P. ovale* subspecies. A TaqMan technology was chosen because the probes provide greater specificity. This method could be used to identify a co-infection between *P. o. curtisi* and *P. o. wallikeri.* So, these TaqMan markers with standard conditions could be added to the set of other markers for the detection of all species in one run. This method was clearly capable of to distinguishing the two *P. ovale* forms.

Ten different genes have been used across the different studies to characterize the 216 *P. ovale* samples. [4,11,18-22]. Two sequences were obtained for each gene and defined two groups of alleles without mixing. The lack of recombination between these two groups of gene supports the notion of separated species. This view is in agreement with the results obtained by the sequencing and the TqPCR.

In 2010, Sutherland described the distribution of the two forms according to country origin [4]. The authors demonstrated that *P. ovale* was divided into two non-recombining sympatric species in Africa and Asia for 55 samples. To complete these data, the study of *P. o. curtisi* and *P. o. wallikeri* in Africa, especially in the Ivory Coast and in the Comoros Islands, was introduced. It complete and deepen the knowledge of the distribution of the two subspecies worldwide (Table 3). 

**Table 3:** **Meta-analysis of *P. ovale *repartition**

**Countries**	***P. ovale***	***P. o. curtisi***	***P.o. wallikeri***	**Author**
Ivory Coast	33	25	8	*
Ivory Coast	1	0	1	[4]
Cameroon	5	4	1	*
Cameroon	1	0	1	[4]
Benin	2	2	0	*
Benin	1	0	1	[4]
Ghana	1	0	1	[22]
Ghana	3	2	1	[4]
Uganda	3	1	2	[4]
Uganda	30	11	19	[21]
Uganda north	6	5	1	[21]
Congo	1	1	0	[4]
Congo Brazzaville	6	2	4	[21]
Nigeria	20	12	8	[4]
Comoros Islands	18	7	11	*
Thailand	10	0	10	[4]
Guinea-Bissau	4	4	0	[4]
Equatorial guinea	4	2	2	[21]
Sierra Leone	3	2	1	[4]
Sao tome	3	2	1	[4]
Chad	2	1	1	*
Burkina Faso	1	1	0	*
Sri-Lanka	1	1	0	*
Tanzania	1	1	0	[4]
Mozambique	1	1	0	[4]
Vietnam	1	0	1	[4]
Papua New Guinea	1	0	1	[4]
Bangladesh	24	11	13	*
Uncertain	1	1	0	[4]
Unknown	23	15	8	*
Multi-countries	5	3	2	*
Total *	90	59	31	*
Total	216	117	99	all

* This study.

The meta-analysis of all the data available indicates that the two subspecies are present in several analysed countries. Nevertheless, more detailed studies must be carried out to specify this data. By extrapolation, two subpopulations are suspected to be present in all countries where *P. ovale* rages if the data was sufficient. Among all the samples included in the study, no co-infection has been detected according results of previous studies [4,21,22]. Recently only one mixed infection of *P. o. curtisi* and *P. o. wallikeri* was described [26].

One important question is the origin of this sympatry. To explain that, Oguike [21] put forward a hypothesis of the parasite’s evolution, the blood polymorphism host group, or a different recognition of certain molecules involved in the parasite cycle. Another hypothesis should be that different *Anopheles* species are present in the same area but at different periods of time. The human malaria vectors being described and present in the Ivory Coast and Comoros Islands are the same mosquitoes species (*Anopheles gambiae* and *Anopheles funestus*) [27,28]. The correlation between the different seasons and the emergence of different malaria vectors should be investigated. This point cannot be explained in this study, because the number of samples is not large enough and too disparate for a given country and a given period of time. Furthermore, varied climatic zones exist within the same country. In order to enlighten this hypothesis, it would be necessary to collect the samples from the same village at the same time. That would allow the comparison of the subtype identification from different climatic periods. Conversely, the repartition of the *P. ovale* subspecies into the various populations of *Anopheles* has never been investigated. This marker would be a tool of choice for it.

The unique difference between the two subspecies was the parasitaemia level. Indeed in one study, *P. o. wallikeri* has been associated with higher levels of parasitaemia in humans [17,18]. In this study, the both parasitaemia averages are very low and similar. The low parasitaemia is often evoked to explain the false negative results in RDT. A recent statement on the eradication programme emphasizes the role of sensitive diagnostic and especially the role of RDT and its lack of performance for neglected malaria, which remains unavailable for the moment in the breakdown of the malaria effort. Nevertheless, the small cohorts of *P. ovale* samples and the different RDT used in the literature make the comparisons difficult between the different studies. The fact that the tests available poorly detect *P. ovale* infection can be the consequence of the genetic difference between the two populations. The RDT must be improved in the light of the information. In the meantime, the specific Tq RT-PCR developed and described here will strongly improve the determination and the characterization of parasitaemia according to the *P. ovale* subspecies.

The *P. ovale* species are considered as neglected. However, severe cases pertaining to acute respiratory symptoms have been reported [6,29-31]. Other severe cases describe spleen rupture [32,33]. These cases have never been the subject of molecular investigation to identify the *P. o. curtisi* or the *P. o. wallikeri* subtype. Taking this parameter into account would be helpful for the knowledge of clinical survey differences possibly existing between the two strains, which could further indicate whether one of the two species is more aggressive. To verify this hypothesis it will be necessary to upgrade this genotyping tool onto a true molecular diagnostic to replace the former *P. ovale* marker.

## Conclusion

The use of the specific primers and probes described here could be a very accurate, useful, easy and rapid method of genotyping two different *P. ovale* subspecies. This technique will allow a better understanding of their characteristics, their biology and their responsibility in the clinical symptomatology. Molecular diagnostics are therefore essential for this purpose.

This study, due to the analysis of a large number of samples, confirms the presence of two subspecies in the same country and increases the knowledge of the two distinguished subspecies through African countries. Finally the question of the existence of six *Plasmodium* species infecting humans could be asked.

## Abbreviations

IC: Ivory Coast; LDH: Lactate dehydrogenase; RDT: Rapid diagnostic test; SSU rRNA: Small subunit ribosomal RNA; TqPCR: Real-time Taqman PCR.

## Competing interests

The authors declare that they have no competing interests.

## Authors’ contributions

FB, CF and JD performed the sequencing and carried out the molecular genetic studies. FB and JD performed the RDT and designed the probes. DP and JD conceived and funded the project. FB and JD prepared the first draft of the paper and all authors contributed to the writing of the report and have reviewed and approved the final version.

## Supplementary Material

Additional file 1 **Alignment of PocLDH and PowLDH.** Using PoLDH (Genbank AY486058) as reference, obtained sequences for *P. o. curtisi* (Poc_LDH) and *P. o. wallikeri* (Pow_LDH) were aligned in nucleotide (A) and translated protein (B) with CLUSTAL 2.0.12 multiple sequence alignment software. The Sequence PocLDH and PowLDH were obtained from samples Po22 and Po23 respectively. In A, the reference sequence AY486058 is related to *P. o. curtisi*. Differences in Pow sequences are box-shaded in grey. In (B), the full sequence of Pf-pLDH protein was added like another reference (Pf13_141). The amino-acid switch between Poc and Pow sequences are indicated with their amino-acid number. All other differences are box-shaded, conservative in grey and non-conservative in white. Click here for file

Additional file 2 **Detail of the sequencing results per sample.** The genotyping result is indicated for each sample (*P. o. curtisi* (C) and *P. o. wallikeri (W)*) with reference sequences obtained by *ldh* or *SSuRNA* and results of POCPOW marker. Corresponding parasitaemia and RDT results according to the antigen used are indicated. neg: RDT negative result; pos: RDT positive result, T2: pan signal. Click here for file
