# Supplementary material for: Real-time PCR assay for discrimination of Plasmodium ovale curtisi and Plasmodium ovale wallikeri in the Ivory Coast and in the Comoros Islands
Source: Malar J. 2012 Sep 4;11:307. doi: 10.1186/1475-2875-11-307 (PMC3489513; doi:10.1186/1475-2875-11-307)
Supplement: Additional file 1 — Alignment of PocLDH and PowLDH. Using PoLDH (Genbank AY486058) as reference, obtained sequences for P. o. curtisi (Poc_LDH) and P. o. wallikeri (Pow_LDH) were aligned in nucleotide (A) and translated protein (B) with CLUSTAL 2.0.12 multiple sequence alignment software. The Sequence PocLDH and PowLDH were obtained from samples Po22 and Po23 respectively. In A, the reference sequence AY486058 is related to P. o. curtisi. Differences in Pow sequences are box-shaded in grey. In (B), the full sequence of Pf-pLDH protein was added like another reference (Pf13_141). The amino-acid switch between Poc and Pow sequences are indicated with their amino-acid number. All other differences are box-shaded, conservative in grey and non-conservative in white. [file 1475-2875-11-307-S1.pdf]

A

```
PoLDH_AY486058   GTTCTCGTTGGTCAGGAATGATAGGAGGTGTTATGGCCACCTTAATTGTTCAGAAAAATT
Poc_LDH          GTTCTCGTTGGTCAGGAATGATAGGAGGTGTTATGGCCACCTTAATTGTTCAGAAAAATT
Pow_LDH          GTTCTCGTTGGTCAGGAATGATAGGAGGTGTTATGGCCACCTTAATTGTTCAGAAAAATT
                 ************************************************************

PoLDH_AY486058   TAGGCGATGTTGTTATGTTCGATATTGTGAAGAACATGCCTCTCGGAAAAGCACTCGACA
Poc_LDH          TAGGCGATGTTGTTATGTTCGATATTGTGAAGAACATGCCTCTCGGAAAAGCACTCGACA
Pow_LDH          TAGGCGATGTTGTTATGTTCGATATTGTGAAGAACATGCCTCTTGGAAAAGCACTTGACA
                 ******************************************* *********** ****

PoLDH_AY486058   CATCACATACAAATGTAATGGCATACTCGAATTGTCAAGTTACGGGATCGAACACATATG
Poc_LDH          CATCACATACAAATGTAATGGCATACTCGAATTGTCAAGTTACGGGATCGAACACATATG
Pow_LDH          CATCACATACGAATGTAATGGCATACTCGAATTGTCAAGTTACGGGATCGAATACATATG
                 ********** ***************************************** *******

PoLDH_AY486058   AGGACTTGAAAGGTGCTGATGTAGTGATAGTAACTGCAGGCTTTACGAAAGCTCCTGGAA
Poc_LDH          AGGACTTGAAAGGTGCTGATGTAGTGATAGTAACTGCAGGCTTTACGAAAGCTCCTGGAA
Pow_LDH          AGGACTTGAAAGGTGCTGATGTAGTGATAGTAACAGCAGGTTTTACGAAAGCTCCAGGAA
                 ********************************** ***** ************** ****

PoLDH_AY486058   AAAGTGACAAAGAATGGAACAGGGATGACTTATTACCATTGAATAACAAAATTATGATTG
Poc_LDH          AAAGTGACAAAGAATGGAACAGGGATGACTTATTACCATTGAATAACAAAATTATGATTG
Pow_LDH          AAAGTGACAAAGAATGGAACAGGGATGACTTATTACCACTGAATAACAAAATTATGATCG
                 ************************************** ******************* *

PoLDH_AY486058   AAATAGGTGGACATATAAAGAATTATTGCCCAAACGCATTTATTATTGTTGTAACTAACC
Poc_LDH          AAATAGGTGGACATATAAAGAATTATTGCCCAAACGCATTTATTATTGTTGTAACTAACC
Pow_LDH          AAATAGGTGGACATATTAAGAATTATTGCCCAAACGCATTTATTATTGTTGTAACTAACC
                 **************** *******************************************

PoLDH_AY486058   CAGCGGATGTTATGGTTCAATTATTACATCAACATTCAGGTGTCTCAAAAAATAAAATTG
Poc_LDH          CAGTGGATGTTATGGTTCAATTATTACATCAACATTCAGGTGTCTCAAAAAATAAAATTG
Pow_LDH          CAGTGGATGTTATGGTTCAATTACTACATCAACATTCAGGTGTCCCAAAAAATAAAATTG
                 *** ******************* ******************** ***************

PoLDH_AY486058   TTGGTTTAGGAGGTGTTCTTGATACATCTAGACTGAAATATTACATTTCTCAAAAATTAA
Poc_LDH          TTGGTTTAGGAGGTGTTCTTGATACATCTAGACTGAAATATTACATTTCTCAAAAATTAA
Pow_LDH          TTGGTTTAGGAGGTGTTCTTGATACATCTAGATTGAAATATTACATTTCTCAAAAATTAA
                 ********************************* **************************

PoLDH_AY486058   ATGTTTGTCCAAGGGATGTAAATGCACATATTGTTGGAGCACATGGCAATAAAATGGTTG
Poc_LDH          ATGTTTGTCCAAGGGATGTAAATGCACATATTGTTGGAGCACATGGCAATAAAATGGTTG
Pow_LDH          AGGTATGCCCAAGGGATGTAAATGCACATATTGTTGGAGCACATGGCAATAAAATGGTTG
                 * ** ** ****************************************************

PoLDH_AY486058   TGTTGAAGAGGTACATAACTGTAGGTGGTATTCCCTTACAAGAATTTATTAACAATAAAA
Poc_LDH          TGTTGAAGAGGTACATAACTGTAGGTGGTATTCCCTTACAAGAATTTATTAACAATAAAA
Pow_LDH          TGTTGAAGAGGTACATAACTGTAGGTGGTATTCCCTTACAAGAATTTGTTAACAATAAAA
                 *********************************************** ************

PoLDH_AY486058   AAATTACAGATGCAGAACTAGATGCCATTTTTGACAGAACTGTTAACACTGCTTTGGAGA
Poc_LDH          AAATTACAGATGCAGAACTAGATGCCATTTTTGACAGAACTGTTAACACTGCTTTGGAGA
Pow_LDH          AAATTACAGATGCAGAACTAGATGCTATTTTTGACAGAACTGTTAACACTGCTTTGGAAA
                 ************************* ******************************** *

PoLDH_AY486058   TCGTTAATTACCATGCCTCTCCATATGTAGCCCCTGCTGCTGCTATTATCGAAATGGCCG
Poc_LDH          TTGTTAATTACCATGCCTCTCCATATGTAGCCCCTGCTGCTGCTATTATCGAAATGGCCG
```

```
Pow_LDH          TTGTTAATTACCATGCCTCTCCATATGTAGCCCCTGCTGCTGCTATTATCGAAATGGCCG
                 * **********************************************************

PoLDH_AY486058   AATCATATCTCAAAGATTTGAAAAAGGTTTTGATATGCTCCACCTTGTTGGAAGGACAAT
Poc_LDH          AATCATATCTCAAAGATTTGAAAAAGGTTTTGATATGCTCCACCTTGTTGGAAGGACAAT
Pow_LDH          AATCATATCTTAAAGATTTGAAAAAGGTTTTGATATGCTCCACCTTGTTGGAAGGACAAT
                 ********** *************************************************

PoLDH_AY486058   ACGGACACACAGGCGTCTTCGGAGGAACGCCTCTCGTCTTGGGATGCAATGGTGTTGAGC
Poc_LDH          ACGGACACACAGGCGTCTTCGGAGGAACGCCTCTCGTCTTGGGATGCAATGGTGTTGAGC
Pow_LDH          ACGGACACACTGGCGTATTCGGAGGAACTCCTCTCGTCTTGGGATGCAATGGTGTTGAGC
                 ********** ***** *********** *******************************

PoLDH_AY486058   AAGTCTTCGAATTGCAGCTAACGCAGAAGAAAAGAAGATGTTTGATGATGCCATT
Pow_LDH          AAGTCTTCGAATTGCAGCTAACGCAGAAGAAAAGAAGATGTTTGATGATGCCA--
Pow_LDH          AGGTCTTCGAATTGCAGCTAACGCAGAAGAAAAGAAGATGTTTGATGATGCCA--
                 * ***************************************************
```

B

```
PoLDH_AY486058   1 --------SRWSGMIGGVMATLIVQKNLGDVVMFDIVKNMPLGKALDTSHTNVMAYSNCQ
Poc_LDH          1 -------RSRWSGMIGGVMATLIVQKNLGDVVMFDIVKNMPLGKALDTSHTNVMAYSNCQ
Pow_LDH          1 --------SRWSGMIGGVMATLIVQKNLGDVVMFDIVKNMPLGKALDTSHTNVMAYSNCQ
PfLDH            1 MAPKAKIVLVGSGMIGGVMATLIVQKNLGDVVLFDIVKNMPHGKALDTSHTNVMAYSNCK
consensus        1         ...*********************.********.*****************.

PoLDH_AY486058  53 VTGSNTYEDLKGADVVIVTAGFTKAPGKSDKEWNRDDLLPLNNKIMIEIGGHIKNYCPNA
Poc_LDH         54 VTGSNTYEDLKGADVVIVTAGFTKAPGKSDKEWNRDDLLPLNNKIMIEIGGHIKNYCPNA
Pow_LDH         53 VTGSNTYEDLKGADVVIVTAGFTKAPGKSDKEWNRDDLLPLNNKIMIEIGGHIKNYCPNA
PfLDH           61 VSGSNTYDDLAGADVVIVTAGFTKAPGKSDKEWNRDDLLPLNNKIMIEIGGHIKKNCPNA
consensus       61 *.*****.**.*******************************************..****

PoLDH_AY486058 113 FIIVVTNPADVMVQLLHQHSGVSKNKIVGLGGVLDTSRLKYYISQKLNVCPRDVNAHIVG
Poc_LDH        114 FIIVVTNPVDVMVQLLHQHSGVSKNKIVGLGGVLDTSRLKYYISQKLNVCPRDVNAHIVG
Pow_LDH        113 FIIVVTNPVDVMVQLLHQHSGVPKNKIVGLGGVLDTSRLKYYISQKLKVCPRDVNAHIVG
PfLDH          121 FIIVVTNPVDVMVQLLHQHSGVPKNKIIGLGGVLDTSRLKYYISQKLNVCPRDVNAHIVG
consensus      121 ********.************* ****.******************.*************
                                         S143P                   N168K

PoLDH_AY486058 173 AHGNKMVVLKRYITVGGIPLQEFINNKKITDAELDAIFDRTVNTALEIVNYHASPYVAPA
Poc_LDH        174 AHGNKMVVLKRYITVGGIPLQEFINNKKITDAELDAIFDRTVNTALEIVNYHASPYVAPA
Pow_LDH        173 AHGNKMVVLKRYITVGGIPLQEFVNNKKITDAELDAIFDRTVNTALEIVNYHASPYVAPA
PfLDH          181 AHGNKMVLLKRYITVGGIPLQEFINNKLISDAELEAIFDRTVNTALEIVNLHASPYVAPA
consensus      181 *******.***************.***.*.****.***************.*********
                                          I204V

PoLDH_AY486058 233 AAIIEMAESYLKDLKKVLICSTLLEGQYGHTGVFGGTPLVLGCNGVEQVFELQLNAEEKK
Poc_LDH        234 AAIIEMAESYLKDLKKVLICSTLLEGQYGHTGVFGGTPLVLGCNGVEQVFELQLNAEEKK
Pow_LDH        233 AAIIEMAESYLKDLKKVLICSTLLEGQYGHTGVFGGTPLVLGCNGVEQVFELQLNAEEKK
PfLDH          241 AAIIEMAESYLKDLKKVLICSTLLEGQYGHSDIFGGTPVVLGANGVEQVIELQLNSEEKA
consensus      241 ******************************...*****.***.******.*****.***.

PoLDH_AY486058 293 MFDDAI----------
Poc_LDH        294 MFDDA-----------
Pow_LDH        293 MFDDA-----------
```

```
PfLDH          301 KFDEAIAETKRMKALA
consensus      301 .**.*.
```
