# Supplementary material for: Real-time PCR assay for discrimination of Plasmodium ovale curtisi and Plasmodium ovale wallikeri in the Ivory Coast and in the Comoros Islands
Source: Malar J. 2012 Sep 4;11:307. doi: 10.1186/1475-2875-11-307 (PMC3489513; doi:10.1186/1475-2875-11-307)
Supplement: Additional file 2 — Detail of the sequencing results per sample. The genotyping result is indicated for each sample (P. o. curtisi (C) and P. o. wallikeri (W)) with reference sequences obtained by ldh or SSuRNA and results of POCPOW marker. Corresponding parasitaemia and RDT results according to the antigen used are indicated. neg: RDT negative result; pos: RDT positive result, T2: pan signal. [file 1475-2875-11-307-S2.doc]

| Samples | Country | LDH seq | *SSU rRNA* seq | POCPOW TqPCR | Parasitaemia  (%) | RDT result | Antigen |
| --- | --- | --- | --- | --- | --- | --- | --- |
| Po1 | IC | C |  | C |  | neg | aldolase |
| Po2 | Comoro Island | C |  | C |  | neg | aldolase |
| Po3 | ? |  |  | C |  | pos T2 | aldolase |
| Po4 | IC | C |  | C |  | neg | aldolase |
| Po5 | ? |  |  | C |  | pos T2 | aldolase |
| Po6 | IC | C |  | C |  | neg | aldolase |
| Po7 | IC | C |  | C |  |  | aldolase |
| Po8 | IC | C |  | C |  |  | aldolase |
| Po9 | ? |  |  | C |  | pos T2 | aldolase |
| Po10 | ? | C |  | C |  | pos T2 | aldolase |
| Po11 | IC | W | W | W |  | neg | aldolase |
| Po12 | ? | C |  | C |  | neg | aldolase |
| Po13 | Comoro Island | W |  | W |  | pos T2 | aldolase |
| Po14 | IC | W |  | W |  |  | aldolase |
| Po15 | IC | W |  | W |  |  | aldolase |
| Po16 | IC | C |  | C |  | neg | aldolase |
| Po17 | IC | C |  | C | 0,058 |  | aldolase |
| Po18 | Gabon / Djibouti | C |  | C | 0,058 | neg | aldolase |
| Po19 | ? |  |  | W | 0,058 | pos T2 | aldolase |
| Po20 | Comoro Island | C |  | C |  |  | aldolase |
| Po21 | Comoro Island | W |  | W |  |  | aldolase |
| Po22 | IC | C |  | C |  |  | aldolase |
| Po23 | Chad | W |  | W |  | pos T2 | aldolase |
| Po24 | IC | C |  | C |  |  | aldolase |
| Po25 | IC | W |  | W |  | neg | aldolase |
| Po26 | IC |  | C | C | 0,025 | neg | aldolase |
| Po27 | Cameroon | C |  | C | 0,033 | pos T2 | aldolase |
| Po28 | Comoro Island | W |  | W | 0,125 | pos T2 | aldolase |
| Po29 | ? | C |  | C | 0,020 | neg | aldolase |
| Po30 | ? | C |  | C |  | neg | aldolase |
| Po31 | Comoro Island | W |  | W | 0,500 | neg | aldolase |
| Po32 | ? | W |  | W | 0,009 | pos T2 | aldolase |
| Po33 | IC | C |  | C | 0,009 | neg | aldolase |
| Po34 | Comoro Island |  | C | C | 0,060 | pos T2 | aldolase |
| Po35 | IC |  |  | C | 0,017 | neg | aldolase |
| Po36 | IC |  |  | C |  | pos T2 | aldolase |
| Po37 | Comoro Island | W | W | W | 0,042 | neg | aldolase |
| Po38 | Burkina Fasso | C |  | C | 0,042 | pos T2 | aldolase |
| Po39 | Bénin | C |  | C |  | neg | aldolase |
| Po40 | IC |  | C | C | 0,100 | neg | aldolase |
| Po41 | IC | C |  | C | 0,058 | pos T2 | aldolase |
| Po42 | Comoro Island | W | W | W | 0,033 | pos T2 | aldolase |
| Po43 | Comoro Island | W | W | W | 0,065 | pos T2 | aldolase |
| Po44 | IC | C |  | C | 0,100 | pos T2 | aldolase |
| Po45 | Vietnam/Cameroon | C |  | C | 0,010 | neg | aldolase |
| Po46 | ? | C |  | C | 0,050 | neg | aldolase |
| Po47 | ? | W |  | W | 0,083 | pos T2 | aldolase |
| Po48 | Comoro Island |  |  | W | 0,009 | neg | aldolase |
| Po49 | IC |  |  | C | 0,005 | neg | aldolase |
| Po50 | Comoro Island |  |  | W | 0,005 | neg | aldolase |
| Po51 | Sri Lanka |  |  | C | 0,009 | neg | aldolase |
| Po52 | IC | C |  | C | 0,090 | pos T2 | aldolase |
| Po53 | IC |  |  | C | 0,000 | pos T2 | aldolase |
| Po54 | Chad | C |  | C | 0,300 | pos T2 | LDH |
| Po55 | Comoro/ Tanzanie |  |  | W | 0,040 | pos T2 | LDH |
| Po56 | Comoro/ Tanzanie | W |  | W | 0,010 | pos T2 | LDH |
| Po57 | ? | W |  | W | 0,000 | pos T2 | LDH |
| Po58 | ? | W |  | W | 0,000 | pos T2 | LDH |
| Po59 | Comoro Island |  |  | W | 0,017 | neg | LDH |
| Po60 | ? |  |  | C |  | neg | LDH |
| Po61 | Comoro Island |  |  | C | 0,000 | pos T2 | LDH |
| Po62 | ? |  |  | C | 0,170 | pos T2 | LDH |
| Po63 | ? |  |  | W | 0,000 | neg | LDH |
| Po64 | IC |  |  | W | 0,200 | pos T2 | LDH |
| Po65 | Cameroon |  |  | C | 0,090 | pos T2 | LDH |
| Po66 | ? |  |  | C | 0,200 | pos T2 | LDH |
| Po67 | ? |  |  | C | 0,000 | pos T2 | LDH |
| Po68 | Bénin |  |  | C | 0,009 | neg | LDH |
| Po69 | IC |  | C | C | 0,153 | neg | LDH |
| Po70 | IC |  |  | W | 0,083 | neg | LDH |
| Po71 | ? |  |  | C | 0,000 | pos T2 | LDH |
| Po72 | IC |  | C | C | 0,100 | neg | LDH |
| Po73 | ? |  | C | C | 0,010 | neg | LDH |
| Po74 | IVC/CAE/CHA/ZIM |  | C | C | 0,100 | neg | LDH |
| Po75 | Comoro Island |  |  | C | 0,000 |  | LDH |
| Po76 | ? |  |  | W | 0,010 | neg | LDH |
| Po77 | ? | C | C | C | 0,003 | neg | LDH |
| Po78 | IC |  | C | C | 0,010 |  | LDH |
| Po79 | Comoros | C | C | C | 0,000 | neg | LDH |
| Po80 | Cameroon |  | C | C | 0,009 | neg | LDH |
| Po81 | IC | C | C | C | 0,000 | neg | LDH |
| Po82 | IC |  | W | W | 0,000 | pos T2 | LDH |
| Po83 | Cameroon |  | W | W | 0,000 | neg | LDH |
| Po84 | IC |  | W | W | 0,000 | neg | LDH |
| Po85 | ? |  | W | W | 0,000 | pos T2 | LDH |
| Po86 | Comoro Island |  | W | W | 0,000 | pos T2 | LDH |
| Po87 | Comoro Island |  |  | C | 0,000 | neg | LDH |
| Po88 | IC |  | C | C |  |  | LDH |
| Po89 | Cameroon | C | C | C | 0,000 |  | LDH |
| Po90 | IC |  |  | C | 0,08 | neg | LDH |
